# Dataset on the effects of spacing and fruit truss limitation on the growth, yield and quality of open-field tomato plants

**DOI:** 10.1016/j.dib.2020.106183

**Published:** 2020-08-17

**Authors:** Long Thien Tran, Anh Tuan Nguyen, Tuan Thanh Nguyen, Ngoc Thi Pham, Long Tien Nguyen, Linh Duc Nhat Hoang, Duc Van Tran, Minh Hong Nguyen

**Affiliations:** a Department of Plant genetics and breeding, Faculty of Agronomy, Vietnam National University of Agriculture, Hanoi, Vietnam; b Research students, Faculty of Agronomy, Vietnam National University of Agriculture, Hanoi, Vietnam; c High quality research and development center, Faculty of Agronomy, Vietnam National University of Agriculture, Hanoi, Vietnam

**Keywords:** Tomato (*Solanum lycopersicum L.*), High plant density, Tomato fruit truss limitation, Cultivation on time-limiting land resources

## Abstract

This article presents data on the effects of spacing and fruit truss limitation on tomato plant growth, yield and fruit quality. Plants with two, three, and four fruit trusses (T1-T3) were grown in four different spaces (S1-S4) to create 12 treatments. The experiment was conducted on an open field with a randomized complete block design and three replications. Data on fruit quantity, weight, and yield were collected to assess the effects of plant density and fruit truss limitation on tomato fruit produced and marketable fruit produced. This data could help develop a strategy for breeding new tomato cultivars for high density planting on the rice-based rotational crop systems in the Red River Delta of Vietnam and other similar sub-tropical regions.

**Specification table**

**Table:** 

Subject	Agriculture
Specific subject area	Horticulture
Type of data	Table, Figure
How data were acquired	Refractometer digital model PR-32α, ATAGO, Tokyo, Japan; The Statistical tool for Agricultural Research (STAR) software, version 2.0.1 (2014).
Data format	Raw
Parameters for data collection	The experiment was conducted on an open field from 15 October 2019 to 12 February 2020. Plants with two, three, and four fruit trusses (T1-T3) were grown in four different spaces (S1-S4) to create 12 treatments.
Description of data collection	Plant structure data was collected for 10 plants per treatment. Yield components were collected weekly at the pink stage from 8 January 2020 to 12 February 2020. Fruit morphology and quality were measured on 15 random fruits from each experimental plot.
Data source location	Institution: Vietnam National University of Agriculture City/Town/Region: Gia Lam, Ha Noi Country: Vietnam
Data accessibility	With the article

**Value of the data**

- The dataset illustrates the effects of growing density and fruit truss limitation on plant growth, yield components, and fruit quality of tomato on the open field.
- The data could be valuable for researchers studying rotational crop production systems. This dataset also includes data on tomato fruit yield harvested after only two weeks. This significantly shortens the total growing duration and enables tomatoes to be grown in different rotational crop systems with time-limited land resources.
- The data also provides a strategy for breeding new tomato cultivars suitable for very high density growing. These cultivars should have short stem, condensed flower truss, a short ripening duration, and simple leaves. These cultivars could be grown in time-limited lands to increase benefits for farmers by reducing labor and material cost while increasing marketable fruit yield.
- The data supports the rotation of tomato cultivars into a rice-based rotational system between rice seasons in the Red River Delta of Vietnam and similar areas. Tomato rotation should occur during the winter season separating the two main winter seasons, as this season offers cooler temperature, fewer pests and lower rate of diseases.

## 1 Data description

### 1.1 Micro-climate data during experimental period

**Fig. 1:**
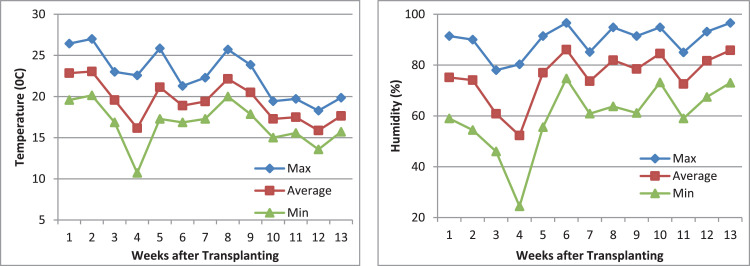
Environmental data (temperature and humidity) during the experimental period

Fig. 1 presents the environmental data on temperature and humidity during 13 weeks following transplantation of tomato plants.

### 1.2 Effects of spacing and truss limitation on plant growth, fruit set, fruit yield, and fruit characteristics

Table 1 presents data on plant height, leaf number and fruit set for each treatment group. The raw data for Table 1 is presented in the Supplementary file “Plant structure and fruit set”

**Table 1:** Effects of spacing and truss limitation on plant structure and fruit set. The data were presented as mean values ± se. Different letters within columns represent statistically significant differences (Tukeys's honest significant difference test, P < 0.05).

Treatment	First Flower Height (cm)	Last Flower Height (cm)	Number of Leaf	Fruit Set (%)
S1T1	53.7 ± 0.1	62.3 ± 0.3^abc^	8.8 ± 0.2^ab^	98.54 ± 1.5
S1T2	50 ± 0.2	69.4 ± 0.4^abc^	8.9 ± 0.3^ab^	96.51 ± 1.4
S1T3	50 ± 2.8	73.0 ± 0.1^ab^	9.6 ± 0.2^ab^	98.41 ± 0.8
S2T1	50.5 ± 2.1	62.6 ± 2.4^abc^	9.1 ± 0.5^ab^	98.66 ± 1.3
S2T2	48.4 ± 2.4	66.9 ± 1.7^abc^	8.6 ± 0.2^ab^	98.38 ± 0.7
S2T3	50.8 ± 1.0	77.2 ± 1.8^a^	10 ± 0.6^ab^	97.71 ± 0.2
S3T1	45.6 ± 1.2	58.0 ± 0.2^bc^	8.3 ± 0.3^b^	99.00 ± 1.0
S3T2	47.1 ± 3.1	68.4 ± 2.2^abc^	8.4 ± 0.1^b^	98.89 ± 1.1
S3T3	50.8 ± 0.6	73.8 ± 4.4^a^	10.3 ± 0.3^ab^	96.58 ± 0.2
S4T1	46.9 ± 1.3	57.5 ± 2.1^c^	8.5 ± 0.1^b^	98.04 ± 0.5
S4T2	47.3 ± 1.5	67.9 ± 3.5^abc^	10 ± 0.6^ab^	97.62 ± 0.5
S4T3	48.6 ± 3.4	69.1 ± 5.5^abc^	10.7 ± 0.5^a^	97.50 ± 0.8
LSD_(0.05)_	ns	15.36	2.18	ns

**Fig. 2:**
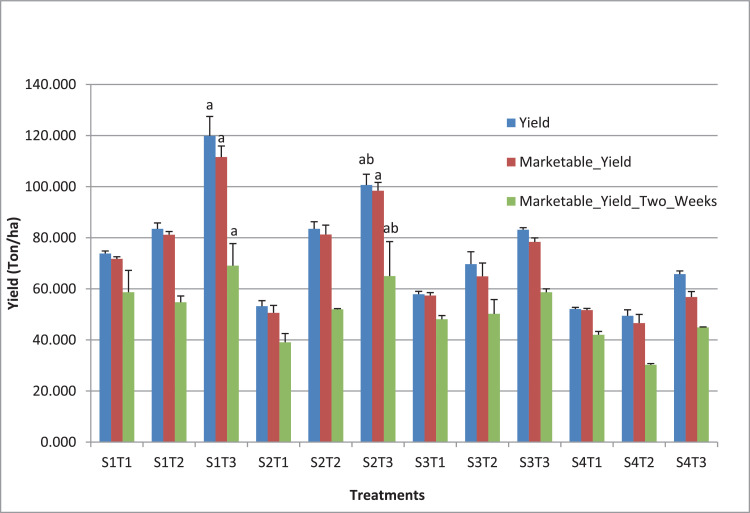
Effects of spacing and truss limitation on tomato fruit yield. The data were presented as mean values ± se. The data for total yield (blue columns) and marketable yield (red columns) are detailed in Table 4; marketable yield harvested after two weeks (green columns) is presented in Table 6. Different letters within a group (by color) represent statistically significant differences (Tukeys's honest significant difference test, P < 0.05).

Data on fruit quantity, fruit weight, and fruit yield were collected to assess the effect of spacing (Table 2), fruit truss limitation (Table 3), and the combination of spacing and fruit truss limitation (Table 4) on total fruit yield and marketable fruit. Table 5 presents the contribution of each fruit truss to total fruit yield and marketable fruit yield. Table 6 shows the effects of spacing and truss limitation on marketable fruit yield harvested in two weeks. Fig. 2 compares the interaction effects of plant spacing and truss limitation on total fruit yield, marketable fruit yield, and marketable fruit yield harvested after two weeks. The raw data for Table 2, Table 3, Table 4, Table 5, and Table 6 is presented in the Supplementary file “Yield components”.

**Table 2:** Effects of spacing on fruit yield components. Different letters within columns represent significant differences (Tukeys's honest statistically significant difference test, P < 0.05). FN: Fruit number, FW: average fruit weight (g), IY: Individual yield (g), Y: Yield (ton/ha).

Spacing	Total fruit				Marketable fruit			
	FN	FW	IY	Y	FN	FW	IY	Y
40 × 30	15.98	88.08	1385.65	92.42^a^	14.75	90.45	1321.82	88.17^a^
40 × 35	15.82	87.15	1385.57	79.11^ab^	15.05	88.82	1344.51	76.77^ab^
40 × 40	16.03	89.29	1404.91	70.25^bc^	14.77	91.87	1337.86	66.89^bc^
40 × 50	16.17	89.19	1394.37	55.78^c^	14.23	92.24	1292.48	51.70^c^
LSD (0.05)	ns	ns	ns	21.23	ns	ns	ns	19.24

**Table 3:** Effects of truss limitation on fruit yield components. Different letters within columns represent statistically significant differences (Tukeys's honest significant difference test, P < 0.05). FN: Fruit number, FW: average fruit weight (g), IY: Individual yield (g), Y: Yield (ton/ha).

Number of truss/ plant	Total fruit				Marketable fruit			
	FN	FW	IY	Y	FN	FW	IY	Y
2 trusses	11.78^c^	95.47^a^	1124.98^c^	59.27^b^	11.28^c^	96.95^a^	1100.40^c^	57.85^b^
3 trusses	15.32^b^	87.42^b^	1336.08^b^	71.53^b^	14.19^b^	90.11^b^	1276.04^b^	68.50^b^
4 trusses	20.90^a^	82.38^b^	1716.82^a^	92.37^a^	18.64^a^	85.47^b^	1596.06^a^	86.30^a^
LSD (0.05)	1.19	6.90	132.62	17.15	1.36	6.01	150.70	17.79

**Table 4:** Effects of spacing and truss limitation on fruit yield components. Different letters within columns represent statistically significant differences (Tukeys's honest significant difference test, P < 0.05). FN: Fruit number, FW: average fruit weight (g), IY: Individual yield (g), Y: Yield (ton/ha).

Treatment	Total fruit				Marketable fruit			
	FN	FW	IY	Y	FN	FW	IY	Y
S1T1	11.8^f^	94.31^abc^	1107.32^de^	73.86^cd^	11.1^ef^	96.28^ab^	1075.41^de^	71.73^bcd^
S1T2	14.4^def^	87.14^bcd^	1251.95^cde^	83.50^bc^	13.75^cde^	88.6^bc^	1217.17^cd^	81.19^bc^
S1T3	21.75^a^	82.78^cd^	1797.68^a^	119.91^a^	19.4^a^	86.47^bc^	1672.88^a^	111.59^a^
S2T1	11.2^f^	83.13^cd^	931.90^e^	53.21^ef^	10.3^f^	85.66^bc^	886.26^e^	50.61^ef^
S2T2	16.1^cde^	90.94^abcd^	1462.18^abc^	83.49^bc^	15.4^bc^	92.37^abc^	1423.67^abc^	81.29^b^
S2T3	20.15^ab^	87.4^bcd^	1762.62^a^	100.65^ab^	19.45^a^	88.42^bc^	1723.58^a^	98.42^a^
S3T1	11.7^f^	99.44^ab^	1157.93^cde^	57.89^def^	11.5^ef^	100^ab^	1147.72^cde^	57.38^def^
S3T2	16.5^bcd^	84.67^bcd^	1393.97^bcd^	69.69^cde^	14.7^cd^	88.98^bc^	1298.22^bcd^	64.91^cde^
S3T3	19.9^abc^	83.77^bcd^	1662.81^ab^	83.15^bc^	18.1^ab^	86.61^bc^	1567.64^ab^	78.38^bc^
S4T1	12.4^ef^	105.03^a^	1302.74^cd^	52.11^ef^	12.2^def^	105.86^a^	1292.18^bcd^	51.69^ef^
S4T2	14.3^def^	86.95^bcd^	1236.21^cde^	49.45^f^	12.9^cdef^	90.47^bc^	1165.12^cde^	46.61^f^
S4T3	21.8^a^	75.58^d^	1644.16^ab^	65.77^cdef^	17.6^ab^	80.38^c^	1420.12^abc^	56.80^def^
LSD (0.05)	3.83	15.75	336.97	19.31	2.85	14.60	304.69	16.32

**Table 5:** Contribution of each truss to fruit yield (ton/ha). The data were presented as mean values ± se.

Treatment	Yield (ton/ha)				Marketable yield (ton/ha)			
	Truss 1	Truss 2	Truss 3	Truss 4	Truss 1	Truss 2	Truss 3	Truss 4
S1T1	39.66 ± 1.12	34.20 ± 2.07			38.63 ± 0.87	33.10 ± 1.71		
S1T2	34.46 ± 0.17	27.28 ± 0.97	21.77 ± 1.50		34.06 ± 0.56	26.65 ± 1.60	20.47 ± 0.20	
S1T3	40.42 ± 5.76	29.33 ± 1.40	25.41 ± 0.45	24.75 ± 0.86	39.30 ± 4.64	27.45 ± 1.16	23.51 ± 2.35	21.33 ± 0.87
S2T1	27.48 ± 3.06	25.73 ± 0.53			27.15 ± 2.67	23.46 ± 0.75		
S2T2	33.77 ± 1.52	25.97 ± 4.89	23.75 ± 3.16		33.77 ± 1.52	25.39 ± 5.56	22.12 ± 2.80	
S2T3	31.73 ± 3.47	28.64 ± 4.55	23.47 ± 1.25	16.81 ± 1.82	31.32 ± 2.99	28.64 ± 4.55	22.79 ± 1.41	15.68 ± 2.35
S3T1	32.43 ± 1.91	25.46 ± 3.42			32.19 ± 2.23	25.19 ± 3.78		
S3T2	28.52 ± 1.82	24.36 ± 2.05	16.82 ± 2.55		27.72 ± 1.34	24.06 ± 1.66	13.13 ± 3.91	
S3T3	26.13 ± 1.02	20.65 ± 0.60	16.39 ± 4.01	19.98 ± 1.32	25.28 ± 0.68	19.54 ± 1.31	14.91 ± 2.29	18.65 ± 2.36
S4T1	27.50 ± 1.34	24.61 ± 0.25			27.08 ± 1.34	24.61 ± 0.25		
S4T2	19.77 ± 3.33	17.27 ± 0.26	12.40 ± 0.33		18.93 ± 3.32	16.17 ± 1.33	11.51 ± 1.03	
S4T3	19.90 ± 0.03	18.93 ± 1.91	15.04 ± 0.03	11.90 ± 0.22	17.11 ± 2.05	17.37 ± 1.64	11.96 ± 3.02	10.37 ± 0.12

**Table 6:** Effects of spacing and truss limitation on marketable fruit yield harvesting in two weeks. Different letters within columns represent statistically significant differences (Tukeys's honest significant difference test, P < 0.05).

Spacing	Number of trusses/plant			Mean
	2 trusses	3 trusses	4 trusses	
40 × 30	58.67^abc^	54.76^bcd^	69.07^a^	60.84^a^
40 × 35	39.08^ef^	51.98^bcde^	64.99^ab^	52.02^a^
40 × 40	48.09^cde^	50.22^cde^	58.66^abc^	52.33^a^
40 × 50	42.04^def^	30.27^f^	44.91^cde^	39.07^b^
Mean	46.97	46.81	59.41	
LSD_(0.05)_ for Spacing = 12.47				
LSD_(0.05)_ for SxT = 13.76

Table 7 presents effects of treatments on fruit morphology (fruit shape index, number of locules, pericarp thickness, and number of seeds per fruit) and fruit quality (^0^BRIX). The raw data for Table 7 is presented in the Supplementary file “Fruit morphology and quality”.

**Table 7:** Effects of spacing and truss limitation on fruit morphology and quality. The data were presented as mean values ± se. Different letters within columns represent significant differences (Tukeys's honest significant difference test, P < 0.05).

Treatment	Fruit shape index	Number of locule	Pericarp thickness (cm)	Number of seed/ fruit	Total soluble solid content (^0^Brix)
S1T1	0.93 ± 0.03	3.35 ± 0.05	0.73 ± 0.04^ab^	140.15 ± 10.25	4.24 ± 0.09
S1T2	0.91 ± 0.01	3.20 ± 0.20	0.76 ± 0.01^a^	144.60 ± 13.90	4.50 ± 0.17
S1T3	0.93 ± 0.03	3.40 ± 0.10	0.74 ± 0.02^ab^	124.89 ± 6.11	4.07 ± 0.20
S2T1	0.93 ± 0.01	3.25 ± 0.05	0.73 ± 0.01^ab^	158.20 ± 20.20	4.63 ± 0.11
S2T2	0.93 ± 0.01	3.60 ± 0.01	0.75 ± 0.01^a^	147.30 ± 8.40	4.24 ± 0.34
S2T3	0.90 ± 0.01	3.40 ± 0.20	0.68 ± 0.02^ab^	159.15 ± 3.05	4.46 ± 0.03
S3T1	0.93 ± 0.02	3.25 ± 0.15	0.72 ± 0.01^ab^	141.55 ± 4.35	4.32 ± 0.20
S3T2	0.94 ± 0.03	3.15 ± 0.05	0.74 ± 0.01^ab^	138.95 ± 2.15	4.53 ± 0.06
S3T3	0.95 ± 0.01	3.45 ± 0.25	0.71 ± 0.04^ab^	132.65 ± 1.95	4.40 ± 0.05
S4T1	0.95 ± 0.01	3.30 ± 0.01	0.72 ± 0.01^ab^	136.35 ± 1.45	4.38 ± 0.01
S4T2	0.92 ± 0.01	3.50 ± 0.10	0.65 ± 0.01^ab^	150.05 ± 4.05	4.20 ± 0.34
S4T3	0.96 ± 0.01	3.15 ± 0.15	0.63 ± 0.01^b^	143.05 ± 1.50	4.14 ± 0.02
LSD_(0.05)_	Ns	ns	0.11	ns	Ns

### 1.3 Table and Figure are presented in “2: Experimental design, materials, and methods”

Table 8 presents data on spacing and fruit truss limitation.

**Table 8:** Treatment details. There were four variations on plant spacing (S1–S4) and three variations on the number of fruit trusses per plant (T1–T3).

Spacing treatments			Truss limiting treatments	
Spacing (row x plant) (cm)		Corresponding plant density (plants/ha)	Number of Truss per plant	
S1	40 × 30	66.700	T1	2 trusses
S2	40 × 35	57.100	T2	3 trusses
S3	40 × 40	50.000	T3	4 trusses
S4	40 × 50	40.000		

**Fig. 3:**
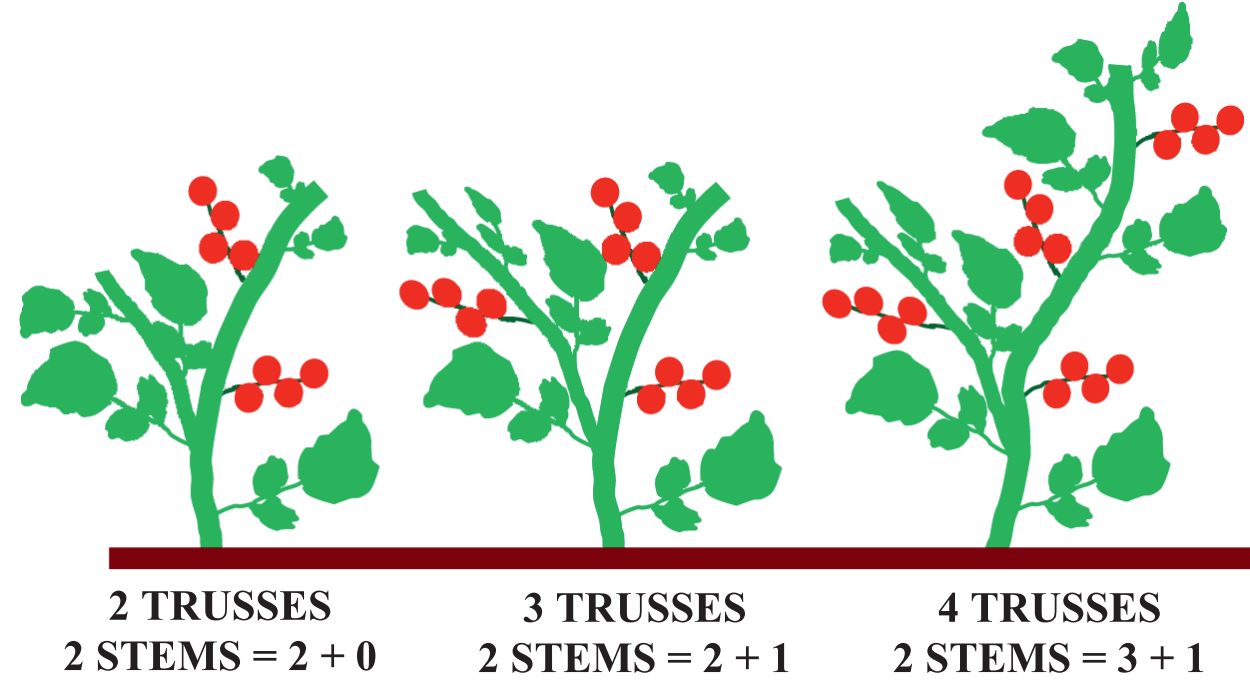
Method for limiting the number of fruit trusses per plant. On all plants, the two main shoots were maintained and two leaves were left above the highest fruit truss. On plants with two trusses, both trusses were on the main shoot; plants with three trusses had two trusses on the main shoot and one truss on the second shoot; plant with four trusses had three trusses on the main shoot and one truss on the second shoot.

Figure 3 illustrates truss limitation and plan morphology for treatment groups.

## 2 Experimental design, materials, and methods

### 2.1 Plant materials and cultivation

The hybrid tomato cultivar VNS585 was provided by Southern Seed Corporation, Vietnam. Seeds were sown on nursery beds inside a net house on 15 October 2019. The 30-day old seedlings were transplanted to the experimental open field. The field was located at the High-Quality Vegetable Research and Development Center (HVRDC) of the Vietnam National University of Agriculture in Hanoi. All agricultural practices related to this experiment, including field cultivation, seedling transplantation, fertilization, irrigation, and other standard agricultural practices were consistent with methods described in Srinivasan's *Safer tomato production techniques* [1].

### 2.2 Experimental design

Twelve treatments were created using four growing spaces and three variations on plant truss (from S1T1 to S4T3). Each plot consisted of two 5-m- long double-rows spaced 1m apart. The 12 plots were grouped in a randomized complete block design with three replications. The details of each treatment are referenced from previous studies [2,3] and appear in Table 8. The highest plant density was 66.700 plant/ha with spacing of 40 × 30 cm (row x plant).

### 2.3 Fruit truss limitation practice

Plants were limited to either 2, 3, or 4 fruit trusses. The lateral branches from the base to immediately below the first flower were removed on all plants. The two main shoots were preserved on all plants, each shoot carrying the number of fruit trusses as indicated in Fig. 3. On all plats, two leaves were maintained above the highest fruit truss [2].

### 2.4 Data collection

Main characteristics related to plant structure (plant height, number of leaves), fruit set, fruit yield components (number of fruits per plant, average fruit weight, fruit yield), fruit morphology (shape, number of locules, pericarp thickness, number of seeds), and fruit quality (^0^Brix) were measured. Plant measurement was collected from 10 plants; fruit data was collected from 15 random fruits. As described previously, data collection was in accordance with practices of the International Plant Genetics Resources Institute [4].

Fruit from each truss was harvested at the pink stage, weighted and recorded weekly for 5 weeks beginning on 8 January 2020. Marketable fruits were defined as fruits weighting 60 g or more. Individual yield was defined as the combined weight of all fruit harvested from each plant. Fruit yield (ton/ha) was calculated by multiplying individual yield by the corresponding plant density for its section. Total Soluble Solid was determined by refractometer (digital model PR-32α, ATAGO, Tokyo, Japan).

### 2.5 Statistical analysis

The Statistical tool for Agricultural Research (STAR) software, version 2.0.1 (2014) was used to conduct an analysis of variance (ANOVA) at P < 0.05; separating mean values by Tukeys's honest significant difference test at P < 0.05.

## Declaration of Competing Interest

The authors declare that they have no known competing financial interests or personal relationships which have, or could be perceived to have, influenced the work reported in this article.

## Appendix Supplementary materials

Image, application 1

Image, application 2

## References

[1] Srinivasan R. (2010). Safer tomato production techniques. AVRDC- World Veg. Cent..

[2] Logendra L.S. (2001). Greenhouse tomato limited cluster production systems: crop management practices affect yield. HortScience.

[3] Jiang C. (2017). Photosynthesis, plant growth, and fruit production of single-truss tomato improves with supplemental lighting provided from underneath or within the inner canopy. Sci. Horticult..

[4] Institute I.P.G.R. (1996).

